# ﻿First record of *Mesoscytina* (Hemiptera, Scytinopteroidea, Scytinopteridae) from the Middle Triassic Tongchuan Entomofauna of China

**DOI:** 10.3897/zookeys.1219.135654

**Published:** 2024-11-27

**Authors:** Qianqi Zhang, Xuheng Du, Xiuping Zhu, Haichun Zhang

**Affiliations:** 1 College of Paleontology, Shenyang Normal University, Shenyang 110034, China Shenyang Normal University Shenyang China; 2 Key Laboratory of Evolution of Past Life in NE Asia, Ministry of Natural Resources, Shenyang 110034, China Ministry of Natural Resources Shenyang China; 3 State Key Laboratory of Palaeobiology and Stratigraphy, Nanjing Institute of Geology and Palaeontology, Chinese Academy of Sciences, Nanjing 210008, China Nanjing Institute of Geology and Palaeontology, Chinese Academy of Sciences Nanjing China; 4 University of Chinese Academy of Sciences, Beijing 100049, China University of Chinese Academy of Sciences Beijing China

**Keywords:** Ladinian, *Mesoscytinatongchuanensis* sp. nov., new record, Scytinopteridae, systematics, taxonomy, Tongchuan Formation

## Abstract

A new scytinopterid species, *Mesoscytinatongchuanensis***sp. nov.**, is established based on a tegmen collected from the Middle Triassic Tongchuan Formation in Shaanxi Province, NW China. The new species can be easily separated from its congeners by the narrow tegminal apex, less curved terminal branches of stems RP, M and CuA and crossvein *r*-*m* connected to long vein M_1+2_. This discovery represents the first record of *Mesoscytina* from the Tongchuan Formation in China and suggests that the genus *Mesoscytina* spread much more widely from Gondwana to northern Pangea in the Middle Triassic.

## ﻿Introduction

The family Scytinopteridae Handlirsch, 1906, an extinct Permian-Triassic hemipteran group widespread in Pangea, is characterized by the costal fracture on the heavily sclerotized punctate tegmen and a hypocostal socket fixed on the thorax (Shcherbakov 1984). Moreover, the basal cell shape, single *r*-*m* crossvein, very weak or absent nodal groove and vein M bent strongly towards CuA are important common characters among Triassic scytinopterid taxa (Lambkin 2016). As ancestors of true bugs, the scytinopteroids are supposed to inhabit temporary submerged waterside vegetation (Shcherbakov 2022). The taxonomic study of Scytinopteridae is of both evolutionary and ecological significance.

The genus *Mesoscytina* Tillyard, 1919 was originally assigned to Scytinopteridae, then to the cercopoid family Archijassidae Becker-Migdisova, 1962 (Hamilton 1992). Lambkin (2016) re-assigned *Mesoscytina* to Scytinopteridae, re-studied the four species of *Mesoscytina* and further proposed the genus *Triassoscarta* being synonymous with *Mesoscytina*. Lara et al. (2021) established the new combination *Mesoscytinaforsterae* (Martins-Neto & Gallego, 2003) (Martins-Neto et al. 2003). Here, we describe a new *Mesoscytina* species from the Tongchuan Entomofauna, which is the fourth Triassic scytinopteroid species discovered in China (Lin 1986; Zhang et al. 2022).

## ﻿Material and methods

The entire tegmen was collected from the Tongchuan Formation of Hejiafang Village, Jinsuoguan Town, Yintai District, Tongchuan City, Shaanxi Province, NW China. A U-Pb geochronology study confirms the insect-bearing layer is dated to 238–237 Ma, as Ladinian of the Middle Triassic (Zheng et al. 2018).

The holotype (NIGP205761) is housed at the Nanjing Institute of Geology and Palaeontology (**NIGPAS**), Chinese Academy of Sciences (**CAS**), Nanjing, China. Photographs were taken using a stereomicroscope system (ZEISS Stereo Discovery V16) in NIGPAS. Images of the part and counterpart of the tegmen were corrected and stacked using Adobe Photoshop 2021, and line drawings were made through software CorelDRAW 2019. The vein nomenclature follows Shcherbakov (1984, 1996). The nomenclatural acts established herein are registered under ZooBank LSID urn:lsid:zoobank.org:pub:72BBDAA4-2F13-4E09-BCF5-C52FB4C81A93.

## ﻿Systematic palaeontology

**Order Hemiptera Linnaeus, 1758**

**Infraorder Cicadomorpha Evans, 1946**

**Superfamily Scytinopteroidea Handlirsch, 1906**

**Family Scytinopteridae Handlirsch, 1906**

### ﻿ Mesoscytina

Taxon classificationAnimaliaHemipteraScytinopteridae

Tillyard, 1919

86BC338A-9881-5C2E-B15A-66BE10719279

#### Type species.

*Mesoscytinaaustralis* Tillyard, 1919.

### ﻿ Mesoscytina tongchuanensis

Taxon classificationAnimaliaHemipteraScytinopteridae

Q. Zhang, Du & H. Zhang sp. nov.

B5BB2A3F-13DB-5645-BCFC-29BD01C4F3E4

https://zoobank.org/871B77B9-0162-476B-B3C9-EE1BA18F3D8A

Figs 1 , 2A

#### Type material.

***Holotype***: No. NIGP205761a, b, an isolated complete tegmen, part and counterpart. Housed at NIGPAS. South of Hejifang Village, Jinsuoguan Town, Yintai District, Tongchuan City, Shaanxi Province, China.

#### Age and horizon.

Ladinian, late Middle Triassic; top of the lower Tongchuan Formation.

#### Etymology.

The specific epithet is from the city of Tongchuan, where the holotype was collected.

#### Diagnosis.

Small tegmen (6–11 mm in length), punctate, quite broad (length/width ratio less than 2.5), broadest on its middle area, with apical area contracted; postcostal area wide; costal fracture curved and single; vein R with two terminal branches; stem M partly curved, with three terminal branches, vein M_1+2_ long; end of vein CuA_2_ beyond vein CuP; terminal branches of stems RP, M and CuA not more or less parallel; crossvein *cua*-*cup* long, less curved veins M_3+4_ and CuA_1_ smoothly connected at crossvein *m*-*cu.* Clavus developed and convex, veins Pcu and A1 forming a “Y” fork; color patterns small and irregular.

**Figure 1.**
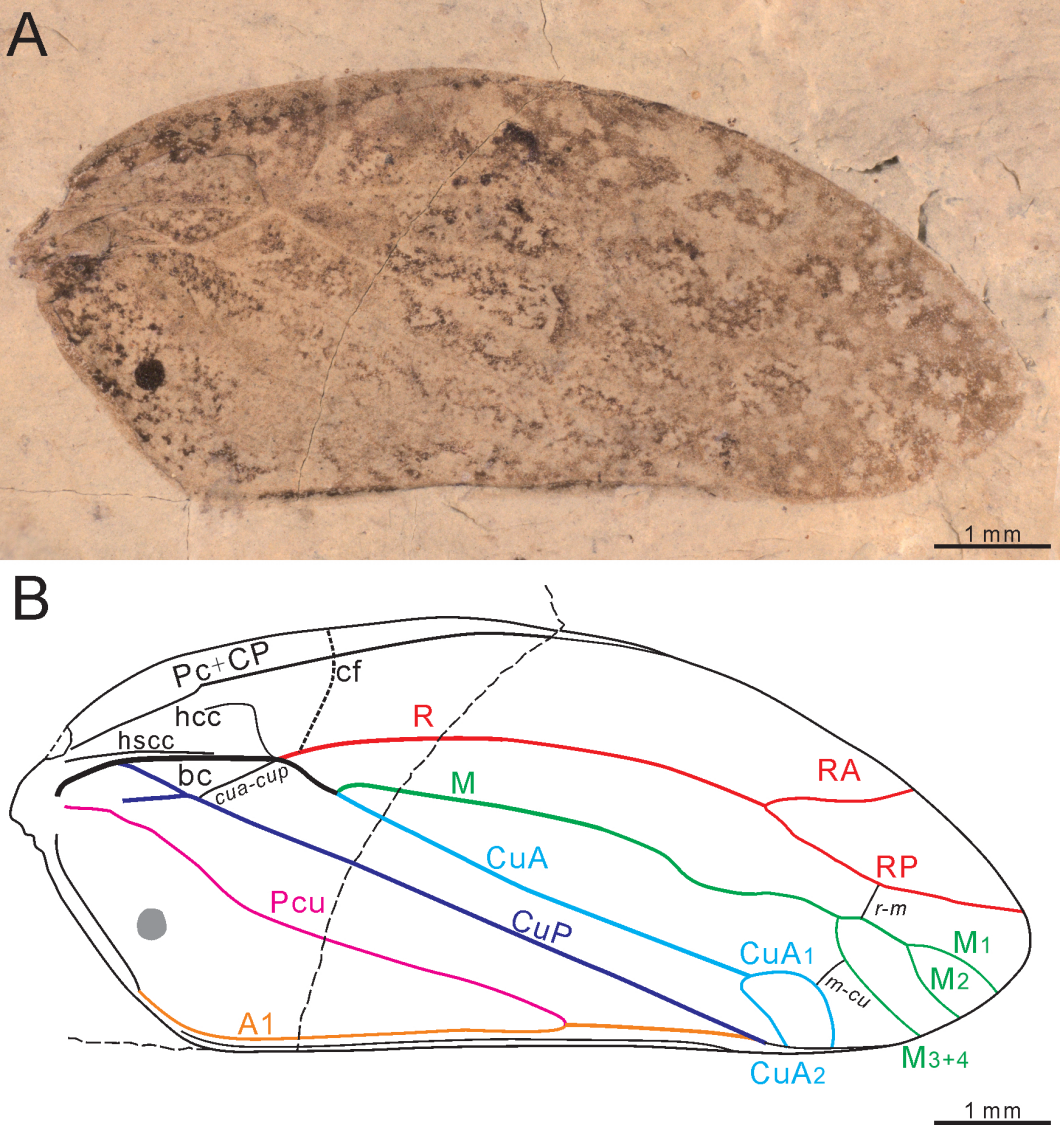
*Mesoscytinatongchuanensis* Q. Zhang, Du & H. Zhang, sp. nov., holotype (NIGP205761), tegmen **A** photograph **B** reconstruction and venation (granules ignored).

#### Description.

***Tegmen*** convex with clavus attached, punctate more distinct on basal tegmen surface. Tegmen length 8.9 mm, maximum width 3.75 mm, length/width ratio 2.37. Tegmen apex asymmetric, gradually narrowed posteriorly. Costa anterior strongly arched, posterior margin more or less straight; tegmen broadest near middle part. Vein Pc+CP curved and merged into anterior margin at basal 3/5 tegmen length. Costal area widest near basal cell; postcostal area much wider than costal area. Hypocostal carina sigmoid, merged into stem R+M+CuA, hyposubcostal carina straight, close to stem R+M+CuA gradually; costal fracture curved apically, originating from stem R, stretched cross vein Pc+CP vertically. Basal cell closed, nearly triangle in shape. Vein R slightly curved, first forked into veins RA and RP at apical 1/4 of tegmen length; vein RA shorter than vein RP in length; vein RP curved posteriorly. Stem M+CuA strong and short, bifurcated first near basal 1/3 of tegmen length. Vein M curved anteriorly first, extending straight on middle membrane, then strongly bent towards vein CuA before reaching level of stem R forking. Vein M first forked into veins M_1+2_ and M_3+4_ at apical 1/5 tegmen length; veins M_1_, M_2_ and M_3+4_ single; crossvein *r*-*m* connected veins RP and M_1+2_; crossvein *m*-*cu* connected to evenly curved M_3+4_ and CuA_1_. Vein CuA single and straight, extending in direction of stem M+CuA; vein CuA divided into veinlets CuA_1_ and CuA_2_ near same level of vein R fork; vein CuA_1_ curved strongly towards posterior margin, vein CuA_2_ shorter than vein CuA_1_, merely just extending beyond clavus apex. Vein CuP single, slightly curved basally before leaving basal cell, then extending straight towards clavus apex after connecting with long crossvein *cua*-*cup.* Clavus large and convex, with anal angle about 120˚; vein Pcu single, curved posteriorly, forming a “Y” fork with vein A1. Vein A2 close to A1, parallel with postclaval margin and merged before end level of vein CuP. Small, irregular, dark-colored patterns shown on tegmen.

**Figure 2.**
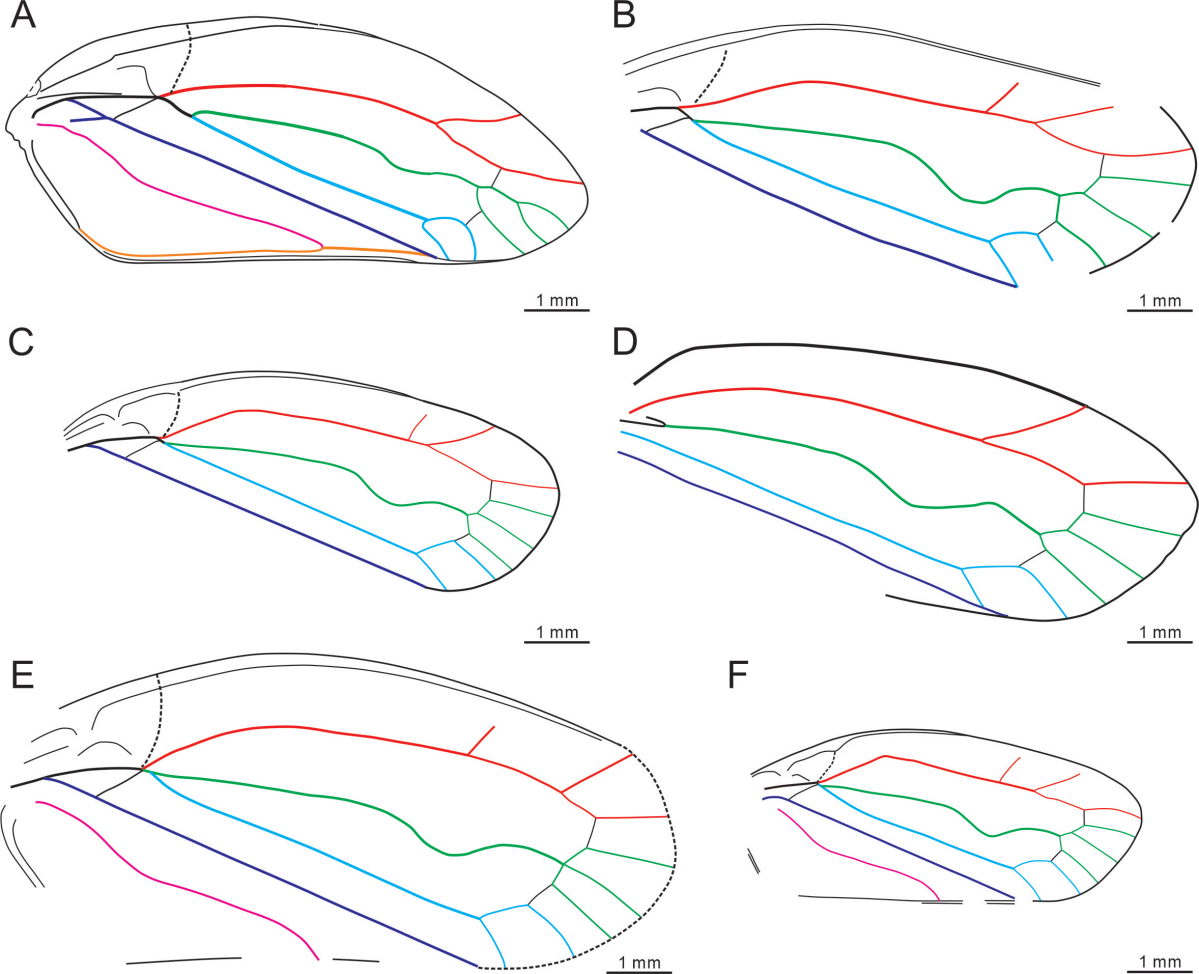
Comparison of tegminal venation in *Mesoscytina* Tillyard, 1919 **A***M.tongchuanensis* Q. Zhang, Du & H. Zhang, sp. nov. **B***M.australis* Tillyard, 1919 **C***M.fistulae* Lambkin, 2016 **D***M.forsterae* (Martins-Neto & Gallego) Lara et al. 2021**E***M.magna* Lambkin, 2016 **F***M.woodsi* Lambkin, 2016.

## ﻿Discussion

According to the key to genera of Scytinopteridae proposed by Zhang et al. (2022), the new species is placed within the genus *Mesoscytina* Tillyard, 1919 by the punctate tegmen, curved vein M, apically forked vein R and three terminal branches of vein M.

So far, there are only five species of *Mesoscytina* Tillyard, 1919 (*M.australis* Tillyard, 1919, *M.fistulae* Lambkin, 2016; *M.forsterae* (Martins-Neto & Gallego, 2003) Lara et al. 2021; *M.magna* Lambkin, 2016 and *M.woodsi* Lambkin, 2016) reported from the Triassic at Mount Crosby, Denmark Hill, Gayndah and Dinmore (Queensland, NE Australia) (Tillyard 1919; Lambkin 2016) and of Tongchuan (Shaanxi, NW China). The notable differences between *Mesoscytinatongchuanensis* Q. Zhang, Du et H. Zhang, sp. nov. and its congeners lie in a lower length/width ratio (at least 2.6 in its congeners), a much longer length of stem M_1+2_, apical terminals of RP, M and CuA less parallel, crossvein *r*-*m* connected to vein M_1+2_ and the apical shape of the tegmen.

Specifically, the new species (Fig. 2A) roughly differentiates from *M.australis* Tillyard, 1919 (Fig. 2B) by a wider but shorter costal area, a narrower apex and less developed R branches. It can also be easily distinguished from *M.fistulae* (Fig. 2C) by the more curved branches of veins M and CuA; from *M.forsterae* (Fig. 2D) in the presence of a larger basal cell, and crossvein *r*-*m* connected to vein M_1+2_ (to M_1_ in *M.forsterae*); from *M.magna* (Fig. 2E) by the shorter tegmen, the less developed areola postica (wider than medial area cells in *M.magna*), three M branches that forked twice (forked into three branches at the same level in *M.magna*); and from *M.woodsi* (Fig. 2F) in having a larger tegmen with base of vein R slightly curved (angulate at base in *M.woodsi*).

## ﻿Conclusion

As the first record of *Mesoscytina* from the Middle Triassic Tongchuan Entomofauna in China, *M.tongchuanensis* Q. Zhang, Du & H. Zhang, sp. nov. is unique by its lateral narrowed apex, less curved terminal branches of stems RP, M and CuA and crossvein *r*-*m* connected to vein M_1+2_. Its establishment not only extends the palaeogeographical record of *Mesoscytina* Tillyard, 1919 from Gondwana to northern Pangea supercontinents of the Northern Hemisphere in the Middle Triassic (Ladinian) but provides the most complete tegminal venation information (especially the clavus) in this genus to date.

## Supplementary Material

XML Treatment for Mesoscytina

XML Treatment for Mesoscytina tongchuanensis

## ﻿Additional information

### Author ORCIDs

Qianqi Zhang https://orcid.org/0000-0001-9354-4610

Xuheng Du https://orcid.org/0000-0002-3476-5074

Xiuping Zhu https://orcid.org/0009-0001-3200-5864

Haichun Zhang https://orcid.org/0000-0003-4553-8317

### Data availability

All of the data that support the findings of this study are available in the main text.

## References

[B1] Hamilton KGA (1992) Lower Cretaceous Homoptera from the Koonwarra Fossil Bed in Australia, with a New Superfamily and Synopsis of Mesozoic Homoptera. Annals of the Entomological Society of America 85(4): 423–430. 10.1093/aesa/85.4.423

[B2] Handlirsch A (1906) Die Fossilen Insekten und die Phylogenie der Rezenten Formen, parts I-IV. Ein Handbuch für Palӓontologen und Zoologen. Verlag von Wilhelm Engelmann, Leipzig, 640 pp.

[B3] Lambkin KJ (2016) Revision of the Scytinopteridae (Hemiptera: Cicadomorpha: Scytinopteroidea) of the Queensland Triassic. Zootaxa 4117: 580–590. 10.11646/zootaxa.4117.4.9 27395195

[B4] Lara MB Bustos-Escalona EL Mancuso AC Arcucci A (2021) Upper Triassic hemipterans from the south-western Gondwana: taxonomical, paleobiological, and paleogeographical implications. Journal of South American Earth Sciences 107(103119). 10.1016/j.jsames.2020.103119

[B5] Lin QB (1986) Early Mesozoic fossil insects from South China. Palaeontologica Sinica (n. s.) Series B, 21. Science Press, Beijing, 112 pp. [in Chinese with English abstract]

[B6] Martins-Neto RG Gallego OF Melchor RN (2003) The Triassic insect fauna from South America (Argentina, Brazil and Chile): a checklist (except Blattoptera and Coleoptera) and descriptions of new taxa. Acta Zoologica Cracoviensia 46(suppl.– Fossil Insects): 229–256.

[B7] Shcherbakov DE (1984) Systematics and phylogeny of Permian Cicadomorpha (Cimicida and Cicadina). Paleontological Journal 1984(2): 87–97.

[B8] Shcherbakov DE (1996) Origin and evolution of the Auchenorrhyncha as shown by the fossil record. In: Schaefer CW (Ed.) Studies on Hemipteran Phylogeny.Entomological Society of America, Lanham, Maryland, 31–45. 10.4182/AMYC5234.1996.31

[B9] Shcherbakov DE (2022) A peculiar new genus of Scytinopteridae (Hemiptera, Cicadomorpha) from the Permian-Triassic boundary beds of Mongolia. Palaeoentomology 5(3): 218–221. 10.11646/palaeoentomology.5.3.2

[B10] Tillyard RJ (1919) Mesozoic insects of Queensland. No. 7. HemipteraHomoptera; with a note on the phylogeny of the Suborder. Proceedings of the Linnean Society of New South Wales 44: 857–896.

[B11] Zhang QQ Zheng DR Teng X Zhang HC (2022) New Scytinopteridae (Hemiptera: Scytinopteroidea) from the middle Triassic Tongchuan Entomofauna of NW China. Historical Biology 34(11): 2259–2264. 10.1080/08912963.2021.2010194

[B12] Zheng DR Chang S-C Wang H Fang Y Wang J Feng CQ Xie GW Jarzembowski EA Zhang HC Wang B (2018) Middle-Late Triassic insect radiation revealed by diverse fossils and isotopic ages from China. Science Advances 4: eaat1380. 10.1126/sciadv.aat1380 PMC6124916 30191177

